# Quercetin Improves Baroreflex Sensitivity in Spontaneously Hypertensive Rats

**DOI:** 10.3390/molecules171112997

**Published:** 2012-11-01

**Authors:** Matheus M. O. Monteiro, Maria S. França-Silva, Naiane F. B. Alves, Suênia K. P. Porpino, Valdir A. Braga

**Affiliations:** Biotechnology Center, Federal University of Paraíba, João Pessoa, PB 58.051-900, Brazil; Email: monteirommo@gmail.com (M.M.O.M.); francasilva_ms@yahoo.com (M.S.F.-S.); naiferraz@gmail.com (N.F.B.A.); suenia_edfisica@yahoo.com.br (S.K.P.P.)

**Keywords:** quercetin, antioxidant, baroreflex, hypertension

## Abstract

Quercetin is a well-known antioxidant. Here, we investigated the effects of treatment with quercetin on mean arterial pressure (MAP), heart rate (HR) and baroreflex sensitivity (BRS) in spontaneously hypertensive rats (SHR). SHR and their controls (WKY) were orally treated with quercetin (2, 10 or 25 mg/kg/day) or saline for seven days. On the 8th day, MAP and HR were recorded. BRS was tested using phenylephrine (8 mg/kg, i.v.) and sodium nitroprusside (25 mg/kg, i.v.). Oxidative stress was measured by tiobarbituric acid reactive species assay. The doses of 10 (n = 8) and 25 mg/kg (n = 8) were able to decrease the MAP in SHR (n = 9) (163 ± 4 and 156 ± 5 *vs*. 173 ± 6, respectively, *p* < 0.05) but not in WKY (117 ± 1 and 118 ± 2 *vs*. 113 ± 1, respectively, *p* < 0.05). The dose of 25 mg/kg/day increased the sensitivity of parasympathetic component of the baroreflex (−2.47 ± 0.31 *vs*. −1.25 ± 0.8 bpm/mmHg) and decreased serum oxidative stress in SHR (2.04 ± 0.17 *vs*. 3.22 ± 0.37 nmol/mL, n = 6). Our data suggest that treatment with quercetin reduces hypertension and improves BRS in SHR via reduction in oxidative stress.

## 1. Introduction

Baroreflex is an autonomic reflex designed to buffer beat-to-beat fluctuations in arterial blood pressure [1]. In addition, hypertension is a strong risk factor for the development of heart failure, myocardial infarction, kidney failure, stroke, and death [2]. This disorder is related to reduction in sensitivity of the baroreceptor reflex. Several studies show that the sensitivity of the baroreflex is diminished in several forms of hypertension [3,4,5]. In addition, several studies show that oxidative stress is a possible cause of hypertension, by a variety of mechanisms [6,7,8,9,10,11]. The imbalance between oxidant and antioxidant species alter the functioning of the central areas involved in blood pressure control [12,13,14,15].

In this context, we highlight the potential of antioxidant substances in the treatment of hypertension. Among them is the flavonol quercetin, a polyphenolic compound found in the human diet and that has its effects by interacting with reactive oxygen species and exerting inhibitory activity against a variety of enzymes, ion channels and transcription factors [15]. Quercetin has been extensively studied in the treatment of hypertension by different mechanisms, including reduction in blood pressure by endothelium-dependent [16] and -independent relaxation [17], decrease in nitric oxide synthase [18], inhibition of vascular superoxide [19] and down-regulation of NADPH oxidase [20]. However, no previous study evaluated the effects of quercetin on baroreflex in spontaneously hypertensive rats.

Therefore, in this study we investigated the effects of quercetin on the depressed baroreflex sensitivity (BRS) in spontaneously hypertensive rats (SHR).

## 2. Results and Discussion

### 2.1. Administration of Quercetin Reduces Blood Pressure in Hypertensive Rats

**Figure 1:**
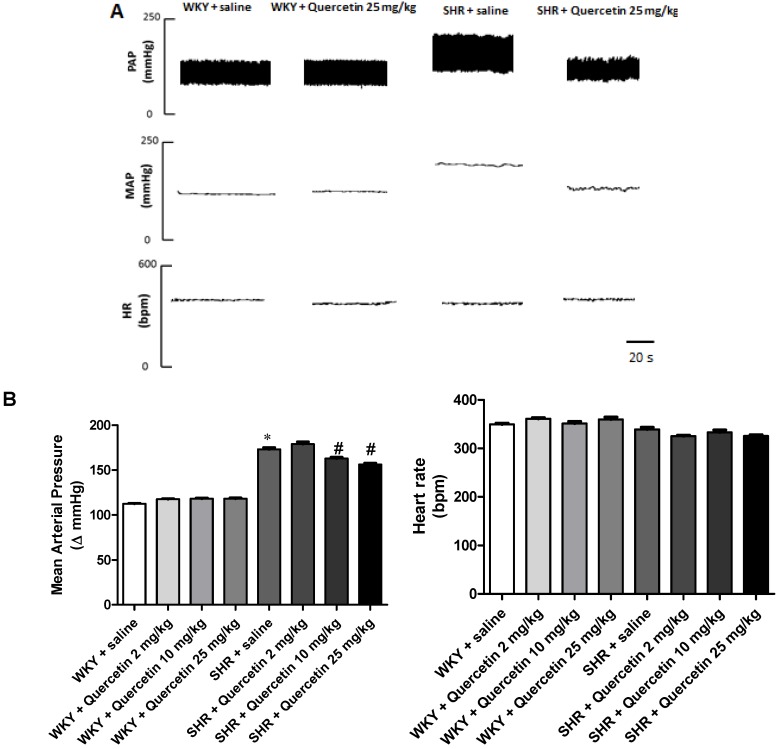
Representative tracings from one rat of each group (WKY + saline, WKY + Quercetin 25 mg/kg; SHR + saline, SHR + Quercetin 25 mg/kg) illustrating the changes in pulse arterial pressure (PAP, mmHg), mean arterial pressure (MAP, mmHg) and heart rate (HR, bpm) (**A**); Effects of Queretin on mean arterial pressure and heart rate (**B**). * *p* < 0.05, when compared to WKY + saline group and ^#^* p* < 0.05 when compared to SHR + saline group.

The Figure 1A shows representative tracing of one animal illustrating changes in pulse arterial pressure (PAP), mean arterial pressure (MAP) and heart rate (HR) in WKY and SHR groups and treated WKY and SHR groups. Cardiovascular parameters were evaluated 24 hours after treatment with quercetin for seven days. Twelve-week-old SHR (n = 9) presented high blood pressure compared to WKY (173 ± 6 *vs*. 113 ± 2 mmHg, respectively, n = 8, *p* < 0.05). Quercetin 2 mg/kg was not able to reduce blood pressure in SHR (179 ± 6 *vs*. 173 ± 5, respectively, n = 7, *p* > 0.05). On the other hand, SHR treated with quercetin 10 mg/kg and quercetin 25 mg/kg (presented reductions in blood pressure when compared to SHR group (163 ± 4 and 156 ± 5 *vs*. 173 ± 6, respectively, n = 8 for each dose, *p* < 0.05). Normotensive rats treated with Quercetin 2, 10 or 25 mg/kg showed no significant changes in blood pressure compared to their controls (117 ± 2, n = 8, 118 ± 2 and 118 ± 2 *vs*. 113 ± 1, respectively, n = 8 for each dose). In addition, there was no significant change in heart rate among groups. These results are shown in the representative tracings and in the group data in Figure 1A and 1B, respectively.

Previous studies demonstrated that quercetin is capable of causing relaxation of peripheral vessels mainly causing decrease in systolic blood pressure in several models of hypertension via its antioxidant capability [18,19,21,22]. Other studies found decrease in systolic, diastolic and mean blood pressure of subjects with hypertension [23] but not pre-hypertensive and normotensive subjects after supplementation with quercetin by 28 days. Oppose to our data, Carlstrom *et al*. reported similar blood pressure between SHR treated with quercetin and SHR compared with WKY rats [24]. Such discrepancy may be due to difference length of treatment and age of rats between studies. It is important to consider that we used 12 week-old SHR and this strain is known to reach its full hypertension around 16 weeks old.

Herein we demonstrated that treatment with quercetin for 7 days produced a 10%-reduction in mean blood pressure. Similar to our data, other study in SHR documented that treatment with quercetin for five weeks (10 mg/kg/day) reduced blood pressure around 21%, but had no effect in normotensive animals [16]. In addition, data from the literature indicates that there is no difference between the oral administration of quercetin as a single or divided into two daily doses [25]. A recent review reported the possible mechanisms that may be responsible for the antihipertensive effect of quercetin such as reduction in oxidative stress, inhibition of angiotensin-converting enzyme activity, and improvement of endothelium-dependent and -independent function. This hypotensive effect of quercetin was demonstrated in animal models of hypertension and in hypertensive patients [23]. The differences between doses may be responsible for different mechanisms of action of this antioxidant; however, this hypothesis is matter for further investigation.

### 2.2. Administration of Quercetin Improves Baroreflex Sensitivity in Spontaneously Hypertensive Rats

**Figure 2:**
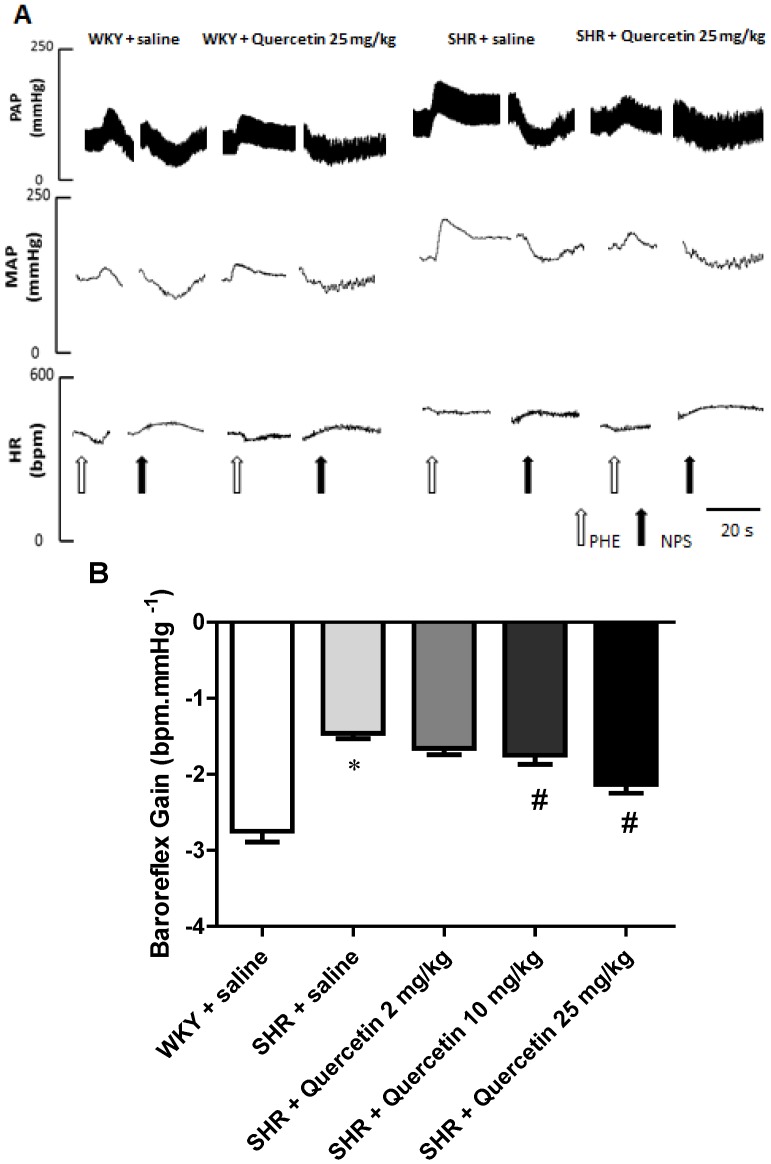
Baroreflex sensitivity test. Representative tracings of a rat from each group (WKY + saline, WKY + Quercetin 25 mg/kg; SHR + saline, SHR + Quercetin 25 mg/kg) illustrating the changes in pulse arterial pressure (PAP, mmHg), mean arterial pressure (MAP, mm Hg), and heart rate (HR, bpm) in response to phenylephrine (PHE, 8 μg/kg—open arrows) and sodium nitroprusside (SNP, 25 μg/kg—black arrows) (**A**). Determination of baroreflex sensitivity. Values for baroreflex gain (bpm/mmHg) determined by the modified Oxford method using bolus intravenous injection of vasoactive drugs sodium nitroprusside (25 μg/Kg) and phenylephrine (8 μg/Kg) in WKY + saline (n = 8, open bar), SHR + saline (n = 9, light grey bar); SHR + Quercetin 2 mg/kg (n = 7, medium grey bar); SHR + Quercetin 10 mg/kg (n = 8, dark grey bar) and SHR + Quercetin 25 mg/kg (n = 8, black bar) groups. (B). * *p* < 0.05, when compared to WKY + saline group and ^#^* p* < 0.05, when compared to SHR + saline group.

BRS of animals was evaluated by intravenous injection of phenylephrine (Phe, 8 mg/kg) and sodium nitroprusside (SNP, 25 mg/kg). Acute administration of Phe was used to evaluate the parasympathetic component and SNP to evaluate the sympathetic component of the baroreflex. Representative tracings of the responses to these drugs are shown in Figure 2A. SHR exhibited a decrease in BRS compared with WKY (−2.75 ± 0.14 *vs*. −1.46 ± 0.07 bpm/mmHg, respectively, *p* < 0.05). The treatment with quercetin at the doses of 10 mg/kg and 25 mg/kg improved in BRS in SHR (−1.75 ± 0.12 and −2.13 ± 0.12 *vs*. −1.46 ± 0.07 bpm/mmHg, respectively, *p* < 0.05), while the dose of 2 mg/kg had no effect (−1.66 ± 0.08 *vs*. −1.46 ± 0.07 bpm/mmHg, respectively), as shown in the Figure 2B. Quercetin produced no important changes in BRS in WKY of any of the doses (data not shown).

**Figure 3:**
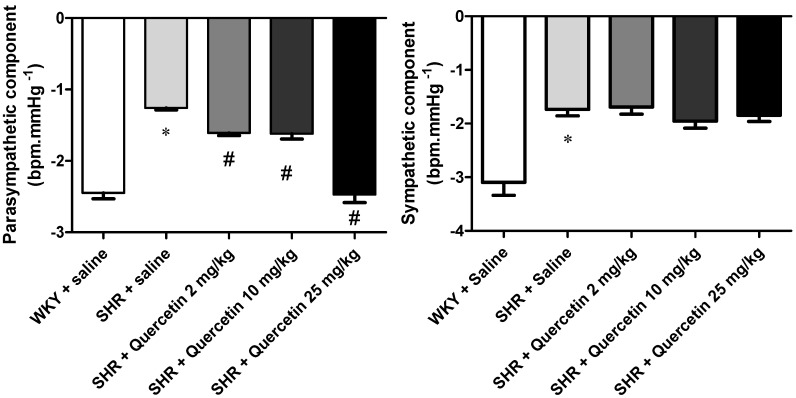
Parasympathetic and sympathetic components of the baroreflex in WKY + saline (n = 8, open bar), SHR + saline (n = 9, light grey bar); SHR + Quercetin 2 mg/kg (n = 7, medium grey bar); SHR + Quercetin 10 mg/kg (n = 8, dark grey bar) and SHR + Quercetin 25 mg/kg (n = 8, black bar) groups. * *p* < 0.05, when compared to WKY + saline group and ^#^* p* < 0.05, when compared to SHR + saline group.

We have previously documented that the BRS is decreased in hypertensive rats in this and other models of hypertension [2,3]. It has been widely reported in the literature that SHR shows sympathetic hyperactivity [26,27]. Analyzing the components of the baroreflex separately, it was also found that quercetin restores BRS in SHR by improving the response of the parasympathetic component being more effectively at the highest dose (−1.60 ± 0.1, n = 8, −1.61 ± 0.2 and 2.47 ± 0.3, n = 8 *vs*. −1.25 ± 0.8 bpm/mmHg, n = 9, *p* < 0.05) (Figure 3). On other hand, quercetin was not able to restore the reduction in the sympathetic component observed in SHR in none of the doses studied (−1.69 ± 0.1, n = 9, −1.95 ± 0.1, n = 6, −1.84 ± 0.1, n = 7 *vs*. −1.73 ± 0.1 bpm/mmHg, n = 9, *p* > 0.05) (Figure 3). There were no changes in treatments with quercetin in normotensive rats in both components (data not shown). Kezeli *et al.* evaluated the influence of quercetin on BRS in rats with myocardial infarction and demonstrated that quercetin increased the reduced BRS resulted from myocardial infarction [28]. However, this is the first report to document the effects of quercetin on baroreflex sensitivity in hypertensive rats.

Although it is not possible to determine the precise mechanism by which our antioxidant therapy with quercetin exerts its positive influence on baroreflex function in spontaneously hypertensive rats, we recently documented that the antioxidant-mediated improvement in baroreflex sensitivity observed in renovascular hypertensive rats is caused by improved autonomic function [3]. Additionally, evidence from other animal studies has suggested that diminished baroreflex sensitivity is caused by endothelial dysfunction [29]. In particular, in experimentally induced endothelial dysfunction, the decrease in prostacyclin and the increase in thromboxane concentrations are associated with reduced baroreflex impulses from the carotid artery [30]. Moreover, experimental evidence strongly suggests a direct suppressive influence of reactive oxygen species on baroreceptors (*i.e*., a peripheral site of action) [31]. In addition, the possible central mechanisms by which quercetin acts on the autonomic function, resulting in improvement of the BRS is still matter for further investigation.

### 2.3. Quercetin Reduces Oxidative Stress in Hypertensive Rats

Spontaneously hypertensive rats present reduced BRS and there is considerable evidence that the mechanisms underlying this reduction may involve oxidative stress [31]. It is known that oxidative stress in the central regions plays a critical role for cardiovascular regulation in SHR [32]. We examined whether treatment with the antioxidant quercetin would be able to reverse the oxidative stress in SHR. Because quercetin (25 mg/kg) was the most effective dose affecting blood pressure and baroreflex sensitivity, this dose was chosen for the studies involving oxidative stress. Spectrophotometric detection of the complex formed between the malonaldehyde (MDA) and thiobarbutiric acid (TBA) has been widely used for measuring lipid oxidation in biological tissues [33].

**Figure 4:**
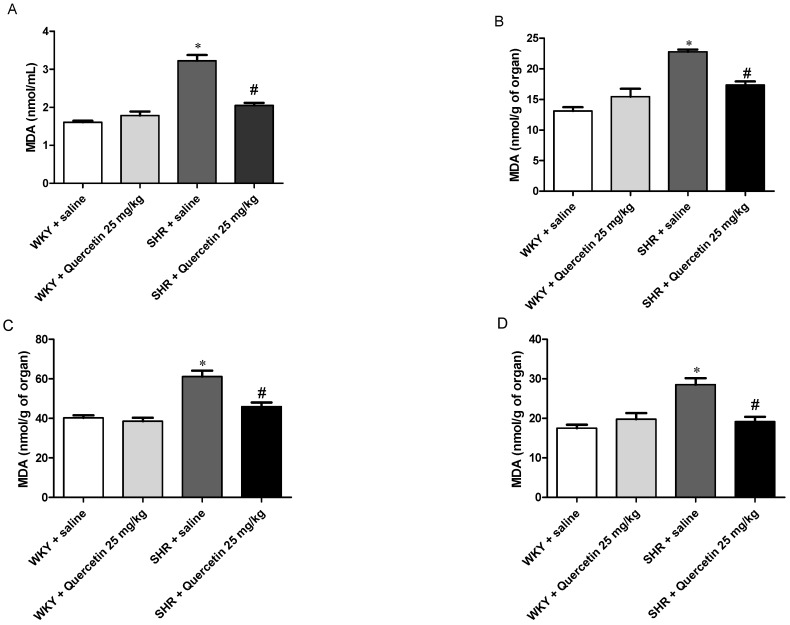
Lipid peroxidation (TBARS) in serum and organs of all four groups (WKY + saline, WKY + Quercetin 25 mg/kg; SHR + saline, SHR + Quercetin 25 mg/kg). Serum levels of TBARS (**A**); TBARS in the heart (**B**); TBARS in the kidney (**C**); TBARS in the liver **D**). * *p* < 0.05, when compared to WKY + saline group and ^#^* p* < 0.05, when compared to SHR + saline group.

The basic principle of the method is the reaction of 1 molecule of MDA and 2 molecules of TBA to form a pink pigment MDA-TBA complex, which can be quantitated spectrophotometrically [34]. In Figure 4A–D, it is possible to observe that quercetin does not affect oxidative stress in normotensive rats and reduces the high levels of oxidative stress found in hypertensive rats.

We found that quercetin improves oxidative stress in several organs related to the cardiovascular system and in the liver, where quercetin is metabolized in one or more metabolites including isorhamnetin, kaempferol, and tamarixetin. In all organs from SHR examined we found increased in oxidative stress and such increase was reversed by treatment with quercetin. On the other hand, WKY presented no significant changes in oxidative stress. These results can be better observed in Table 1 and Figure 4.

**Table 1:** Determination of oxidative stress using the thiobarbituric acid-reactive substances (TBARS) assay in organs and serum of all four groups (WKY + saline, WKY + Quercetin 25 mg/kg; SHR + saline, SHR + Quercetin 25 mg/kg).

	Serum (nmol/mL)	n	Heart (nmol/g)	n	Liver (nmol/g)	n	Kidney (nmol/g)	n
WKY + Saline	1.6 ± 1.1	9	13.13 ± 1.5	7	17.5 ± 2.1	6	40.2 ± 3.0	5
WKY + Quercetin (25 mg/kg)	1.78 ± 0.2	4	15.45 ± 2.5	4	19.79 ± 3.3	5	38.55 ± 4.7	7
SHR + Saline	3.2 ± 0.3 *	6	22.78 ± 0.9 *	6	28.51 ± 3.9 *	6	61.13 ± 7.3 *	6
SHR + Quercetin (25 mg/kg)	2.04 ± 0.2 ^#^	6	17.33 ± 1.6 ^#^	7	19.19 ± 2.6 ^#^	5	45.98 ± 4.1 ^#^	4

* *p* < 0.05, when compared to WKY + saline group and ^#^* p* < 0.05, when compared to SHR + saline group.

Flavonoids, including quercetin, can interfere with ≥3 different free radical–producing systems, among them: direct radical scavenging, decrease high concentrations of nitric oxide produced by inducible nitric-oxide synthase in macrophages, inhibit xanthine oxidase activity that is a source of oxygen free radicals, decrease the number of immobilized leukocytes to the endothelial wall that is another major mechanism responsible for the formation of oxygen-derived free radicals. Interaction with other enzyme systems and chelation of iron, which in combination with reactive oxygen species, results in lipid peroxidation [35].

Duarte *et al*. showed that quercetin restores the increase of MDA in liver of SHR, but do not change liver glutathione peroxidase and glutathione reductase activities *in vitro*, maybe because quercetin is acting by mechanisms other than increased activity of antioxidant enzymes. However, it can be acting by scavenging free radicals [16]. In previous studies the liver MDA was decreased in the liver of rats but not in humans supplemented with quercetin and this can be explained by differences in plasma levels of the latter substance [23]. Nevertheless, Carlstrom confirmed that quercetin increases the levels of MDA increased in SHR as compared with the WHY [24].

It is known that hypertension is associated with renal and heart injury, we investigated the oxidative stress in the kidney. Our results show that the quercetin was able to decrease the levels of TBARS increased in SHR. According Liu and colleagues, quercetin is able to inhibiting of kidney inflammation that trigger renal injury due at least in part to its anti-oxidant activity and its ability to modulate the mitogen-activated protein kinases (MAPKs) and nuclear factor kappa B (NF-κB) signaling pathway [36]. Meanwhile, Zhang *et al*. confirmed the protective effects of quercetin in liver, kidney but not in heart by iron creators effects in iron-overloaded mice. In heart flavonoids were more efficient in inhibiting the increase of carbonyl content and the TBARS levels [37].

Taken together these data demonstrate that quercetin acts systemically reducing oxidative stress, probably by scavenging free radicals and improving the baroreflex sensitivity in hypertensive rats.

## 3. Experimental

### 3.1. Animals and Treatment

Forty-eight adult male SHR and Forty-five male WKY rats (270–320 g) were housed in a temperature-controlled room set to a 12:12-hour light-dark cycle with free access to standard rat chow (Labina®, Purina, Paulinea, SP, Brazil) and water, were used. When the animals completed 12 weeks, they were treated with a daily dose of quercetin (2, 10 and 25 mg/kg, v.o. by gavage) or Saline for seven days. Animals were divided in eight different groups: WKY + Saline, WKY + Quercetin 2 mg/kg, WKY + Quercetin 10 mg/kg, WKY + Quercetin 25 mg/kg, SHR + Saline, SHR + Quercetin 2 mg/kg, SHR + Quercetin 10 mg/kg, SHR + Quercetin 25 mg/kg. All procedures described in the present study are in agreement with Institutional Animal Care and Use Committee of the Federal University of Paraiba (CEPA/LTF protocol n 0407/11).

### 3.2. Blood Pressure and Heart Rate Recordings

One day before the experiments, rats were anesthetized with ketamine and xylazine (75 and 10 mg/kg, both i.p., respectively) and fitted with femoral venous and arterial catheters for drug injection and arterial pressure recordings, respectively. Blood pressure measurements were performed ≥24 hour after catheter implantation as previously described [13]. Blood pressure and heart rate were recorded in conscious rats using a pressure transducer (MLT0380/D, ADInstruments, Sydney, Australia) and connected to a computer (Mikro-tip Blood pressure system, ADInstruments, Australia) running the LabChart software (ADInstruments, Australia).

### 3.3. Baroreflex Sensitivity Test

At the day of the experiment, following baseline blood pressure and heart rate recordings, baroreflex was activated using the classical vasoactive drugs phenylephrine (8 mg/Kg) and sodium nitroprusside (25 mg/Kg) (modified Oxford Method, Braga *et al*.) [38]. Phenylephrine was used to evaluate the parasympathetic component of the cardiac baroreflex while sodium nitroprusside was employed to evaluate the sympathetic component of the cardiac baroreflex. All data from each group were analyzed by linear regression using the GraphPad Prism software and the slope of the linear regression yield baroreflex gain for each group.

### 3.4. Tiobarbituric Acid Reactive Species (TBARS) Assay

Serum, heart, liver and kidney samples were collected in all groups. The lipid peroxidation level in samples was measured as MDA, which is the end product of lipid peroxidation, and reacts with TBA as a TBARS to produce a red colored complex which has peak absorbance at 532 nm as described previously [39]. In brief, the organs were washed with saline to minimize the interference of hemoglobin with free radicals and to remove blood adhered to the tissues. The organs were homogenized to 10% of tissue with potassium phosphate buffer. Then, 250 mL was removed and stored at 37 °C for 1 hour, after which 400 mL of 35% perchloric acid was added, and the mixture was centrifuged at 14,000 rpm for 20 minutes at 4 °C. The supernatant was removed, mixed with 400 mL of 0.6% thiobarbituric acid and incubated at 60 °C for 1 hour. After cooling, the absorbance at 532nm was measured. A standard curve was generated using 1,1,3,3-tetrametoxypropane. The results were expressed as nmol of MDA/mg of tissue for organs and as nmol of MDA/ml for serum.

### 3.5. Statistical Analyses

Results are expressed as mean ± SEM. Data were analyzed by Student’s t test or two-way repeated measures analysis of variance (ANOVA) followed by a Dunnett post-test for multiple comparisons whenever appropriate. All statistical analyses were performed using GraphPad Prism (v. 5.0, GraphPad Software Inc., San Jose, CA, USA). Statistical significance was defined as *p* < 0.05.

## 4. Conclusions

In summary, our results suggest that antioxidant therapy by treatment with quercetin improves baroreflex sensitivity in spontaneously hypertensive rats. Despite the precise site of action where the anti-oxidant treatment produces its beneficial effects remains unknown, it seems to involve both peripheral and central mechanisms, since quercetin not only improved oxidative stress in serum, heart, liver and kidneys, but also improved the parasympathetic component of the baroreflex.
